# Care-seeking behavior of Japanese gynecological cancer survivors suffering from adverse effects

**DOI:** 10.1186/1472-6874-13-1

**Published:** 2013-01-08

**Authors:** Sumiko Oshima, Kengo Kisa, Takayoshi Terashita, Hidenobu Kawabata, Masaji Maezawa

**Affiliations:** 1 Department of Healthcare Systems Research, Graduate School of Medicine, Hokkaido University, Sapporo, Japan; 2 Department of Psychology and Communication, School of Humanities, Hokusei Gakuen University, 2-3-1 Oyachi-nishi, Atsubetsu-ku, Sapporo, 004-8631, Japan; 3 Faculty of Health Sciences, Hokkaido University, Sapporo, Japan

**Keywords:** Health behavior, Adverse effects, Gynecological cancer, Quality of health care, Post-treatment follow-up

## Abstract

**Background:**

Post-treatment follow-up visits for gynecological cancer survivors should provide opportunities for management of adverse physical/psychological effects of therapy and early recurrence detection. However, the adequacy of such visits in Japan is poorly documented. We qualitatively explored care-seeking experiences of Japanese gynecological cancer survivors and deduced factors influencing care-seeking behaviors and treatment access.

**Methods:**

We conducted 4 semi-structured focus groups comprising altogether 28 Japanese gynecological cancer survivors to collect a variety of participants’ post-treatment care-seeking behaviors through active interaction with participants. Factors influencing access to treatment for adverse effects were analyzed qualitatively.

**Results:**

Survivors sought care through specialty clinic visits when regular post-treatment gynecological follow-ups were inadequate or when symptoms seemed to be non-treatment related. Information provided by hospital staff during initial treatment influenced patients’ understanding and response to adverse effects. Lack of knowledge and inaccurate symptom interpretation delayed help-seeking, exacerbating symptoms. Gynecologists’ attitudes during follow-ups frequently led survivors to cope with symptoms on their own. Information from mass media, Internet, and support groups helped patients understand symptoms and facilitated care seeking.

**Conclusions:**

Post-treatment adverse effects are often untreated during follow-up visits. Awareness of possible post-treatment adverse effects is important for gynecological cancer survivors in order to obtain appropriate care if the need arises. Consultation during the follow-up visit is essential for continuity in care.

## Background

Many survivors of gynecological cancer suffer from persistent adverse post-treatment effects [1]. Reported post-treatment symptoms include constipation/diarrhea, lymphedema, menopausal symptoms, sexual and vaginal dysfunction, and neurotoxicity [2-5]. Physical sequelae lead to higher levels of depressive symptoms [6]. In the case of patients undergoing long-term treatment, these symptoms tend to result in lowered quality-of-life and unmet needs for symptom management [7,8].

Effective management of late complications of cancer treatment would contribute to the well-being of cancer survivors [9]. Hence, it is imperative to understand the experiences of gynecological cancer survivors during care for adverse treatment outcomes; unfortunately, this aspect has not been well studied [1]. Routine post-treatment follow-up visits provide gynecological cancer survivors opportunities for management of physical/psychological adverse effects and early recurrence detection [10-12]. However, in Japan, there is sparse documentation of the follow-up experiences of gynecological cancer survivors suffering from prolonged symptoms.

Japan has a universal health insurance system that allows patients to choose any specialty clinic of their choice. All patients are covered under the insurance regardless of their disease. In this system, registration of patients with general physicians and gatekeeping by general physicians are not required; consequently, continuity and comprehensiveness of primary care remain poor [13]. Therefore, post-treatment follow-up is provided usually by cancer specialists. Patients consult cancer specialists directly after the treatment at regular but decreasing intervals over several years. Proposed gynecological cancer follow-up programs in the clinical guidelines are similar to those of Western countries, but these programs are often more frequent and intensive than those of Western countries [14].

Gynecological cancer survivors are usually considered to consult cancer specialists for post-treatment symptoms. However, as mentioned above, Japan’s health insurance system allows cancer survivors to be examined and treated for post-treatment symptoms at any specialist clinic of their choice. Therefore, the purpose of this study was to explore qualitatively the care-seeking experiences, both during and outside of follow-ups, of post-treatment Japanese gynecological cancer survivors suffering from adverse effects. We also examined factors influencing care-seeking behaviors and access to treatment for adverse symptoms.

## Methods

### Participants and data collection

A qualitative approach was chosen for this research for the following reasons: first, qualitative research has advantages for exploring subjective experiences and understanding of the study participants [15], and second, qualitative methods have been used to study care seeking behaviors in published literature [16,17]. Focus groups, a type of group interview, were adopted to promote self-disclosure and active discussion through interaction of a small number of participants [18].

The data used in the study were part of a larger qualitative project that explored the perspectives of post-treatment care of gynecological cancer survivors. We sought gynecological cancer patients on follow-up 1–10 years post-treatment without recurrence by invitation in a newsletter sent to approximately 400 members of a Japanese gynecological cancer support group based in Hokkaido. This recruitment strategy was chosen to collect a wide range of experiences from survivors attending different hospitals. Of the 32 women who showed interest, one cervical cancer survivor who post-treatment period was less than 1 year and two breast cancer survivors were excluded. Before the focus group interviews, a questionnaire was mailed to obtain each participant’s profile (Table 1).

**Table 1:** **Characteristics of study participants** (**n** = **28**)

**Characteristics**	**n**
**Age(years)**	
40–49	7
50–59	7
60–69	13
70–79	1
**Cancer site**	
Cervix	9
Endometrium	11
Ovary	7
Vulva	1
**Time since completion of treatment(years)**	
1–2	5
2–3	3
3–4	3
4–5	6
5–6	1
6–7	4
7–8	3
8–9	0
9–10	3
**Treatment received**	
Surgery alone	12
Surgery and chemotherapy	15
Surgery, chemotherapy, and radiation therapy	1

Four focus groups comprising 6–8 participants each were formed. Semi-structured group interviews were conducted in each group to discuss post-treatment experiences related to follow-up and expectations regarding treatment by health professionals. After the introduction, participants were asked to express their views regarding their experiences and perspectives in post-treatment care and symptoms using the following open-ended questions: (1) what do you think about post-treatment care?; (2) if you had a problem after treatment, was it solved in post-treatment care?; and (3) are you satisfied with post-treatment care and why?. The answers were explored further if participants touched on their experiences of care seeking. The sessions were conducted during November and December 2009 at local community centers in four different regions of Hokkaido. As the participants touched on their experiences while seeking care for adverse effects, the interviewer probed deeper into the answers.

The research protocol for this study was approved by the ethics committee of Hokkaido University Graduate School of Medicine. All participants received detailed information about the study and provided written consent. The principal author (SO) conducted all interviews. Each session lasted 60–90 min and was audio taped with permission and transcribed verbatim. One participant requested that her data be withheld; thus, focus-group data obtained from 28 women were subjected to analysis. The participants were aged 41–71 years. Participants’ cancers included major gynecological malignancies, which helped us to explore the diversity in the care-seeking experiences of gynecological cancer survivors. Nine survivors had cervical cancer, 11 had endometrial cancer, 7 had ovarian cancer, and 1 had vulvar cancer. Post-treatment period was ≤5 years for 17 survivors and 5–10 years for 11 survivors. Twelve survivors had been treated with surgery alone; 15 had received surgery and chemotherapy; and 1 had received surgery, chemotherapy, and radiation therapy. Regarding educational background, 2 survivors had not graduated from high school, 17 were high school graduates, and 9 were college graduates.

### Data analysis

Using thematic analysis, accounts of participants’ care-seeking behaviors were coded and grouped into categories according to commonalities and differences [19]. Relationships among categories were determined by extracting statements defining category–category connections. Data analysis was continued until data saturation, in which no new categories and category–category connections were extracted. The category–category connections were visualized by diagramming care-seeking pathways to treatment and factors influencing the process of receiving treatment, by using an event flow network technique recommended by Miles and Huberman [20].

The supervisor (MM) and other team members (KK, TT, and HK) discussed data validity and altered codes and categories when necessary.

## Results

Participant data revealed care-seeking experiences for 16 types of post-treatment symptoms: lymphedema (16 women), urinary disorder (9), menopausal symptoms (2), fatigue (2), numbness (2), taste disorder (1), pain (1), irritability (1), depression (1), bowel obstruction (1), abdominal pain (1), constipation (1), cystitis (1), palpitation (1), inguinal hernia (1), and disk hernia (2). The description of inguinal hernia and disk hernia refers to symptoms that worsened after treatment.

**Figure 1:**
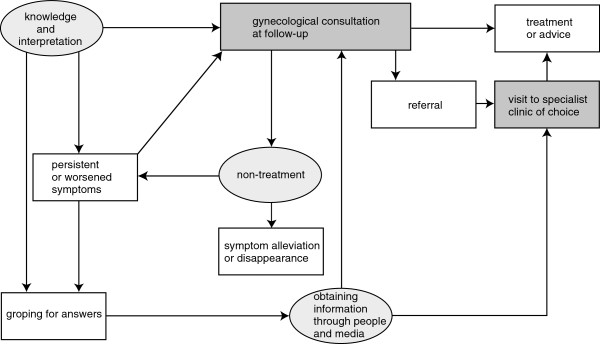
**Care**-**seeking pathways of gynecological cancer survivors with post**-**treatment adverse effects.** The circles show the factors identified in the study that influenced care-seeking behaviors, while the boxes show patients’ care-seeking behaviors, and the arrows indicate the relationships between the factors and behaviors. Highlighted boxes represent the 2 major pathways of care-seeking.

Analysis revealed 10 categories related to careseeking: “knowledge and interpretation,” “persistent or worsened symptoms,” “groping for answers,” “gynecological consultation at follow-up,” “non-treatment,” “symptom alleviation or disappearance,” “referral,” “obtaining information through people and media,” “visit to specialist clinic of choice,” and “treatment or advice.” The study identified 15 types of category–category relationships, as shown in Figure 1.

The category–category relationships revealed that patients receive treatments for adverse effects through 2 pathways: “gynecological consultation at follow-up” and “visit to specialist clinic of choice.” Three factors, namely, “knowledge and interpretation,” “non-treatment,” and “obtaining information through people and media,” were found to directly influence care reception at the 2 pathways leading to treatment. Descriptions of these 2 pathways and 3 factors are given below. The other 5 categories are also indicated in relation to these 2 pathways and the 3 factors.

### Gynecological consultation at follow-up

Among the two observed pathways, gynecological consultation at follow-up visits presented as an important opportunity for symptomatic women to seek care. Ten women described their experience of consulting gynecologists about their post-treatment symptoms at follow-up visits. They told their gynecologists about feelings of discomfort, pain, or symptoms similar to those described to them prior to discharge after initial treatment.

“I felt grumpiness when preparing meals or doing something similarly insignificant so that I even felt like beating on a rice bowl or something…when he asked me how I was doing, I told him about it and that I felt burning and couldn’t sleep. He prescribed me a pill.” (#15, 1 year after treatment)

At follow-up consultations, women experienced 3 types of responses from gynecologists in reaction to their symptoms: (1) “treatment or advice,” (2) “referral,” or (3) “non-treatment.” Treatments received at follow-up included drug prescriptions for menopausal symptoms, urinary disorders, and swollen legs (“*treatment or advice*”). Physicians referred lymphedema patients to outpatient clinics specializing in particular symptoms. Urinary disorders, menopausal symptoms, and disk/inguinal hernia patients received recommendations to visit other departments or local clinics (“*referral*”). “It was about half a year after the treatment when I told my doctor that I felt something was wrong. He said “I expected that” and recommended that I buy a special stocking…but the symptom didn’t get better. In the next visit, the doctor recommended that I visit a lymphedema clinic.” (#11, 6 year after treatment)

One woman told that she had consulted her doctor but was unsure about the timing of treatment. “When I told my doctor at follow-up about inguinal hernia, he said that he could refer me to a specialist. However, sometimes the discomfort continues and sometimes it disappears. So I waited while wondering whether someday I will have to receive surgery.” (#10, 5 years after treatment)

### Visit to specialist clinic of choice

In addition to follow-ups, women sought care at nearby clinics and from departments other than gynecology at the hospitals where they received cancer treatment. A total of 10 experiences were described by 6 women. They visited 5 types of clinical doctors; internists, psychiatrists, urologists, lymphedema specialists, and acupuncture therapists. Women cited 3 reasons for visiting doctors outside of follow-up: (1) they did not think that their symptoms were related to cancer treatment, (2) they did not receive treatment at follow-ups, and (3) they thought that there was no point in consulting the gynecologist at follow-up.

One woman said she visited a nearby urologist when she experienced urine leakage after treatment. There, she received information for the first time that her symptom was a result of cancer treatment. “I felt something was wrong and visited a urologist… I understood what was happening when he told me it was because of the surgery…he was the first person to tell me that gynecological surgery could disrupt the bladder.” (#26, 4 years after treatment) She added that she was not informed that the surgery for cancer treatment could cause urinary disorder.

Another woman who received no treatment or instruction when consulting a gynecologist about swollen legs at a follow-up said she visited a lymphedema clinic that she herself had found. “There was no treatment or instruction on how to ease symptoms at that time. So I looked for a lymphedema clinic, visited there on my own judgment, and learned about self-care…I could manage to solve my problems by making my own decision of consulting a specialist.” (#27, 6 years after treatment)

Those who believed that there was no point in consulting the gynecologist at follow-up said that they felt this way because their doctors had changed or because of the hospital’s attitude when they were in-patients. “If you were to see the same doctor who treated you, you might be able to complain or tell him that you ended up having such-and-such symptoms. But, all of the doctors who were in charge of my treatment have left, and I am afraid that there is no point complaining to my current doctor. So I think I have no choice but to see a urologist about urinary problems.” (#14, 7 years after treatment)

Description about 3 factors influencing care reception, “knowledge and interpretation,” “non-treatment,” and “obtaining information through people and media” is given below:

### Knowledge and interpretation

Among factors influencing the treatment process, scarcity of information and incorrect interpretation of the symptoms were the leading factors influencing the lack of need for the women to seek professional care for their adverse effects. Knowledge about adverse effects and interpretations of signs and symptoms affected whether women sought care and how they participated in care. Eleven women said they received information about possible adverse effects by hospital staff before surgery or at discharge. They obtained knowledge through surgical consent forms, pre-operative consultations, and discharge documents. Possible adverse effects mentioned on those occasions included lymphedema, urinary dysfunction, neurotoxicity, and menopausal symptoms. Those who developed urinary dysfunction before discharge also gained knowledge through their treatment experience at that time. The majority of informed women consulted gynecologists at follow-ups when experiencing signs or symptoms. “I was told by the doctor that I would develop lymphedema and visited the clinic referred by the doctor.” (#09, 1 year after treatment)

In contrast, 5 women claimed that they had not received information on adverse effects from hospital staff before discharge. Some of these women visited nearby clinics when symptoms developed, while the others did not even think about seeking care, ignoring their symptoms until they worsened and only later realizing that their symptoms, including depression, lymphedema, and urinary dysfunction, were effects of gynecological cancer and treatment. One woman said she knew nothing about lymphedema even after she first developed the symptom soon after surgery. Unable to realize that her symptom was treatment related, she spent her daily life with prolonged symptoms. Her symptoms were persistent and suddenly worsened (“*persistent or worsened symptoms*”). “I felt tingling pain in my right leg soon after the surgery. I thought it was an effect of anesthesia and would be better soon, but the pain continued. After discharge, I made myself walk around the neighborhood to build up my strength. Soon I found my leg got very swollen. I was so surprised.” (#29, 3 years after treatment) It was at this point that she visited her gynecologist.

Interpretation of symptoms affected whether women sought care or not. Three women who did not seek care accepted the symptoms as signs of old age or a legacy of treatment. Others thought they could manage the symptom or that the symptoms would disappear soon. Some women whose chronic symptoms worsened after treatment did not seek care because the symptom had existed before cancer treatment.

One woman who experienced fatigue but did not seek care voiced her concern about the interpretation of her symptoms. “I wonder where this fatigue comes from. Is it just because I am physically weak or due to the surgery that removed cancer? Does this have anything to do with the removal of lymph nodes? I felt so exhausted after working 4 hours a day and cannot continue working anymore.” (#30, 2 years after treatment)

Many women who realized the connection between symptoms and cancer treatment wished that they had received information earlier. One woman who visited a psychiatrist after a year of worry about depressing feelings said, “After wondering for some time why I felt pains in my chest and back, I thought it might be stress and visited a psychiatrist. He said it could be some kind of psychiatric effect since there were no such symptoms before…the symptom gradually disappeared…If I knew such symptoms would occur after treatment beforehand, I would’ve understood the symptom.” (#26, 4 years after treatment) Here, she also illustrated her days of wondering what to do with prolonged symptoms (“*groping for answers*” and “*persistent or worsened symptoms*”).

### Non-treatment

Women with pain, lymphedema, and neurotoxicity said that their doctors did not provide care for adverse effects at follow-up when they first complained about the symptoms. Among 12 women who claimed of not receiving care at follow-ups, 5 received a diagnosis but did not receive treatment, while 7 received neither a diagnosis nor the treatment. Women’s recollections of their doctors’ explanations for non-treatment included statements such as “the symptom was not serious,” there was “no treatment for the symptom,” or “it would disappear over time.” Some doctors simply said that they had no knowledge of the symptom.

Among those women who were told by gynecologists that their symptom would disappear, one woman experienced remission of the symptom (“*symptom alleviation or disappearance*”). One woman who had a taste disorder after chemotherapy said, “The doctor told me it would disappear over time. I said ‘I see.’ I know there are people whose symptoms did not disappear after several months, but in my case, my taste recovered in a month and a half or so. So, I thought it did recover once the treatment completed.” (#12, 1 year after treatment)

However, there were women whose symptoms continued or worsened (“*persistent or worsened symptoms*”). For example, one woman who suffered numbness in her toes after chemotherapy said she was distressed by the duration of the symptom. She repeatedly complained about the symptom at follow-ups, but her doctor kept telling her to wait. The doctor’s attitude perturbed her. “He said it would disappear soon. However, as time elapsed, he initially said it would take 3 years and then later said 4 years. I wondered whether he didn’t care enough to treat me or didn’t know how to.” (#02, 1 year after treatment)

### Obtaining information through people and media

Symptomatic women who did not interpret the symptom as a result of cancer treatment or who did not receive the treatment while consulting their gynecologists at follow-ups described how they groped for answers to find ways to ease their symptoms (“*groping for answers*”). For those women, information played a key role in obtaining treatment. Those who obtained useful information were able to take a step forward to either “gynecological consultation at follow-up” or “visit to a specialist clinic of choice” while those who did not kept wondering what to do.

Sources of information that were discussed included newspapers, television, the Internet, patient groups, and nurses. The adverse effect most often mentioned with regard to information seeking was lymphedema. One woman recalled finding an article about lymphedema in a newspaper. “I happened to find an article in a medical newspaper that described a doctor who left the university and opened a clinic. With that information, I visited there.” (#28, 7 years after treatment)

The Internet was mentioned as a source of information by patients and their family members. One woman, whose doctor stated that he was not aware of the symptom after the patient complained about her swollen leg at follow-up, said that her daughter discovered online a medical institution for treatment. “My daughter searched the Internet and found a local medical university’s vascular surgery department that appeared to do something…The doctor there said the symptom would last a lifetime and told me to use a special German-made stocking. It was from that doctor that I first understood that I was suffering from lymphedema.” (#13, 9 years after treatment)

Newspaper articles or Internet searches were the source of information on patient groups. One woman mentioned that she immediately contacted a patient group that held regular meetings at a local community center when she first experienced signs of lymphedema. “I met a patient with a swollen leg during my hospital stay. She did not receive any treatment. I thought the hospital would not provide treatment if I developed a symptom, so I searched for a patient group by myself and made a contact.” (#07, 6 years after treatment)

Nurses were unofficial sources of information. Some women spoke to the nurses with whom they had become acquainted during their hospital stay, while others talked with nurse or ex-nurse friends. “My doctor first told me it wasn’t serious and just prescribed Kampo [Chinese herbal medicine]. The symptom was not bad when I saw him because it was always in the morning, but it got worse at night. …it was around that time my ex-nurse friend warned me. She told me I should take care, otherwise it could become serious.” (#12, 1 year after treatment)

After obtaining information on symptoms, women either visited clinics or talked to their gynecologists about the information at the follow-up. For example, one woman realized her leg had swollen when she attended a lecture on lymphedema hosted by a patient group. She consulted a gynecologist at the next follow-up visit, but he provided no treatment. After consultation, she met a nurse she had known since treatment and explained the situation. “I showed my leg to the doctor, but he said ‘Well, anyone’s leg can get swollen this much.’…As I was wondering what to do, I saw a head nurse with whom I talked a lot during my hospital stay…She said ‘It’s better to visit a specialist,’ so I did. My lymphedema turned out to be in an early stage…I think my leg would’ve been much worse if I hadn’t attended the lecture hosted by the patient group.” (#06, 6 years after treatment)

## Discussion

This is the first qualitative study to explore the care-seeking behaviors of Japanese gynecological cancer survivors suffering from post-treatment adverse effects. Although our data are drawn from 28 participants, this study aims to extract variations in care seeking experiences and the sample size was considered to be adequate because the analysis reached data saturation, in which no new categories and category–category connections were extracted [21,22].

The result revealed 2 pathways to treatment (“*gynecological consultation at follow*- *up*” and “*visit to specialist clinic of choice*”) and 3 factors affecting care-seeking behaviors (“*knowledge and interpretation*,” “*non*-*treatment*,” and “*obtaining information through people and media*”).

Survivors used follow-up consultations as opportunities to receive care for adverse effects. Strikingly, however, women’s stories revealed that follow-up consultations did not always result in symptom investigation or treatment. In those cases, symptoms often continued and even worsened. Delays in treatment of adverse effects were associated with the attitudes of gynecologists during follow-up visits, indicating that provider delays affect treatment of survivors suffering from adverse effects, as in cancer diagnosis [23,24].

This study also revealed that gynecological cancer survivors seek care at a variety of clinics outside of follow-up consultations. This is possible in Japan, where patients have a free choice of service providers under the universal health insurance system. In this study, women visited neighborhood clinics of choice when they suspected symptoms not to be related to cancer treatment, when they did not receive care at follow-up, or if they did not have high expectations for a good outcome after gynecological consultation. In these instances, patient delays occurred while they wondered about symptoms and which clinic to visit. The freedom to choose any clinic, and not the one recommended by gynecologists at follow-ups, may bring a lack of continuity in care and a loss of precious information essential for the clinician to guide the patient through treatment.

These observations also strongly indicate that Japanese gynecological cancer survivors have needs for the management of adverse effects. Previous quantitative studies revealed that gynecological cancer survivor with lymphedema have more unmet physical and informational needs and that those with physical sequelae reported lower levels of meaning in life, which was associated with higher levels of depressive symptoms [6,25]. In addition, gynecological cancer survivors were found to have needs for comprehensive cancer care, which include communication with doctors and use of local health care services [3]. Another survey on survivors of various cancer types, including gynecological cancer, reported high needs for and low satisfaction with information on long-term side effects [26]. Taken together, it is suggested that more emphasis should be placed on doctor-patient coordination and provision of care for prolonged post-treatment symptoms of gynecological cancer survivors.

From participants’ accounts shown in the result, they sought care both during and outside of follow-up regardless the time from the treatment completion. The women’s narrative about their symptoms and care-seeking behaviors highlight that not only the type of symptoms but how they seek care affects the outcome and duration of the post-treatment symptoms. It indicates that there are some women who resume normal daily lives without realizing that they may need help for their symptoms caused by cancer and its treatment.

Furthermore, the strong dissatisfaction about the lack of care was expressed by those who finished the treatment relatively recently. There are 2 possible explanations for these observations. One explanation is that the care for post-treatment adverse effects has not been much improved. Those for whom many years have passed since treatment may accept their symptoms or be used to the lack of care. Another explanation is that those for whom the treatment is recent feels stronger care needs for adverse effects.

Information obtained from sources other than medical institutions, including mass media and patient groups, played an important role for patients in reaching proper treatment. The observation that patients did not receive enough information from medical institutions is in accord with a previous study reporting that cancer survivors did not receive information needed to cope with debilitating symptoms [27]. These findings highlight the need to explore the scope and role of follow-up programs for the care of cancer survivors. Other studies have pointed out that cancer survivors do not receive all the information they need about sequelae and their treatment during follow-up care [28,29]. However, a fully developed and standardized model for providing follow-up care service does not exist [1]. In the case of gynecological cancer, follow-ups primarily focus on disease recurrence, and patients consider this aspect of follow-up to be the most important [30,31]. A recurrence-oriented consultation may delay seeking and providing care for adverse effects; this may be an important area of research, to explore the role of follow-up in caring for adverse effects and in providing patients with information on how to obtain appropriate treatment for such effects. Studies focusing on Survivorship Care Plans or on different follow-up care models, as undertaken in other countries, may be a way forward. However, in Japan, open discussion about policies or guidelines for follow-up care is rare. At the very least, medical professionals engaged in follow-up programs should encourage patients to express their symptoms during consultation, and care providers should pay much more attention to the detection and treatment of adverse effects.

Our study also shows that whether and how survivors initiate care-seeking behavior depends on their knowledge and interpretation of their symptoms. Survivors’ knowledge about adverse effects came from consultations before surgery and at the time of discharge. Uninformed survivors often failed to identify and acknowledge the presence of signs and symptoms. They also failed to relate the symptoms they experienced to their cancer treatment. In contrast, informed survivors sought care at follow-up consultations or clinics specializing in treating their symptoms. Studies on delays in cancer diagnosis have previously identified the role of knowledge and interpretation [32-34]. The results of the present study emphasize the importance of raising awareness of adverse effects in gynecological cancer treatment so that survivors interpret signs and symptoms correctly and take timely and appropriate action.

Andersen’s model of total patient delay describes delays caused by both patients and health professionals [35]. This model was proposed for cancer diagnosis and then applied to various other diseases as a framework to explore help-seeking behavior [36]. Delays documented in this study are consistent with what Andersen calls “appraisal,” “illness,” “behavioral,” and “treatment” delays. In the present study, appraisal delay occurred when gynecological cancer survivors failed to interpret signs and symptoms as those requiring medical attention. Illness delay occurred when survivors postponed seeking medical help, and behavioral delay occurred when survivors wondered where to seek help. These delays are patient-related; however, this study suggests that the lack of information provided by healthcare professionals before discharge also contributes to increased delays. Treatment delay occurred when survivors consulted a gynecologist at follow-up but received neither treatment nor referral. Previous studies on help-seeking for cancer symptoms revealed that healthcare professionals’ “poor advice,” non-investigation of symptoms, and lack of referrals caused delays in cancer diagnosis [23,24]. The present study suggests that Andersen’s model could be used to formulate guidelines to improve the treatment of adverse effects in Japanese gynecological cancer survivors.

This study has several limitations. First, because the participants came from a specific patient group, our findings might not be generalized to the cancer survivor population as a whole. Second, there may be a volunteer bias and those who have unsatisfied post-treatment experiences may be more likely to participate in the study. Third, there may be a recall bias and their accounts may not accurately reflect their actual experiences. Fourth, since participants were recruited from different hospitals, their experience may have reflected the local protocols of those hospitals.

In addition, we were not able to confirm delays experienced by survivors who visited a clinic of their choice but did not receive appropriate treatment, although this could fit into the category of treatment delay. Furthermore, we were unable to explore the behaviors of patients after receiving their initial treatment for post-treatment symptoms, and therefore such information is lacking.

Nonetheless, this study offers new and useful insights into care-seeking behaviors and treatment delay after cancer treatment. Cross-sectional surveys of randomly selected survivors will determine the characteristics of those with unresolved survivorship concerns. Furthermore, prospective studies will provide information about the process of post-treatment care-seeking behaviors.

## Conclusion

Awareness of possible adverse effects of both the disease and the treatment is important for gynecological cancer survivors so that they may obtain appropriate care if the need arises. Patients should receive detailed information about the signs and symptoms of these effects at discharge in such a way that they can adequately interpret any developing symptoms. Medical professionals engaged in follow-up programs should routinely encourage patients to express their symptoms and concerns during follow-up consultation in order to make a differential diagnosis of reported symptoms for referrals and/or additional treatment.

## Competing interest

The authors declare that they have no competing interest.

## Authors’ contribution

SO conducted the focus group discussions and the initial data analysis and drafted the manuscript. KK, TT, and HK participated in data analysis. MM checked data validity. All authors read and approved the final manuscript.

## Pre-publication history

The pre-publication history for this paper can be accessed here:

http://www.biomedcentral.com/1472-6874/13/1/prepub
